# A protocol for staged arthroplasty to salvage infected nonunion of hip fractures

**DOI:** 10.1007/s10195-016-0419-6

**Published:** 2016-07-11

**Authors:** Ayman M. Ebied, Adel I. Elseedy, Osama Gamal

**Affiliations:** Orthopaedic Department, Faculty of Medicine, Menoufia University, Gamal Abdel Nasser Street, Shebin El Kom, Menoufia Governorate Egypt

**Keywords:** Hip fracture, Infection, Failed internal fixation, Antibiotic spacer, Total hip arthroplasty

## Abstract

**Background:**

Nonunion of hip fractures is not uncommon. Total hip arthroplasty is used to salvage cases of non union or secondary arthritis in these fractures. However, this option may not be available or may be difficult to achieve when infection has superseded the site of nonunion. The objective of this prospective study was to assess if a staged protocol of treatment yields good results in these difficult cases.

**Materials and methods:**

Twenty-seven consecutive patients who had deep hip infection with failed treatment of hip fractures (intracapsular in 16 cases and extracapsular in 11) were treated between June 2007 and September 2011. Twenty-six completed the planned two-stage hip arthroplasty and one case was lost after the first stage. The average age of the patients was 48.9 years (range 26–74 years) with an average follow up period of 44 months (30–72 months). Analysis was done using the paired *t* test where *P* < 0.05 was considered significant.

**Results:**

Infection was controlled in all cases that completed the treatment protocol with no recurrence in all cases at the latest follow up. The Harris hip score of the patients improved significantly from 29 preoperatively to 85 at the latest follow up (*P* < 0.0001). Two patients had hip dislocation with displacement of the trochanteric fragment while three other patients had fibrous union of the trochanter.

**Conclusions:**

Staged Arthroplasty procedure to salvage infected non-union of hip fractures is successful in eradicating infection and regaining hip function.

*Level of evidence* IV.

## Introduction

Treatment of hip fractures, both the intra and extra-capsular types, remains elusive to orthopaedic surgeons [1]. It is not uncommon to have cases of nonunion and failure of osteosynthesis [2]. Total hip arthroplasty is used in many occasions to salvage cases of nonunion or secondary arthritis in these fractures [3]. However, this option may not be available or may be difficult to achieve when infection has superseded the site of non union.

Although excision arthroplasty known as Girdle Stone procedure has been advocated in medically unfit candidates, it is not a suitable procedure in young or active patients. Staged arthroplasty is an accepted strategy at many centers, but usually associated with high rate of complications and only few reports have been published [4].

In these cases, a priority in management is to eradicate infection which can then be followed by reconstruction. The surgical debridement and identification of the infecting organisms are therefore essential steps.

The difficulties associated with staged arthroplasty are usually related to soft tissue contracture, muscle wasting, acetabular and femoral bone defects, disturbed anatomy of the proximal femur as well as trochanteric reattachment. Therefore, preoperative planning, implants and techniques of revision surgery should be available to the treating surgeon.

In this series, a protocol of staged treatment for cases of infected non union of hip fracture has been employed and prospectively evaluated.

## Materials and methods

In the period between June 2007 and September 2011, in a prospective study, 27 consecutive patients with hip fractures were treated using a staged protocol for hip arthroplasty. Sixteen of these patients had intracapsular fracture while the remaining 11 had extracapsular fracture configuration. Six out of these extra capsular fractures were in the intertrochanteric area.

**Fig. 1:**
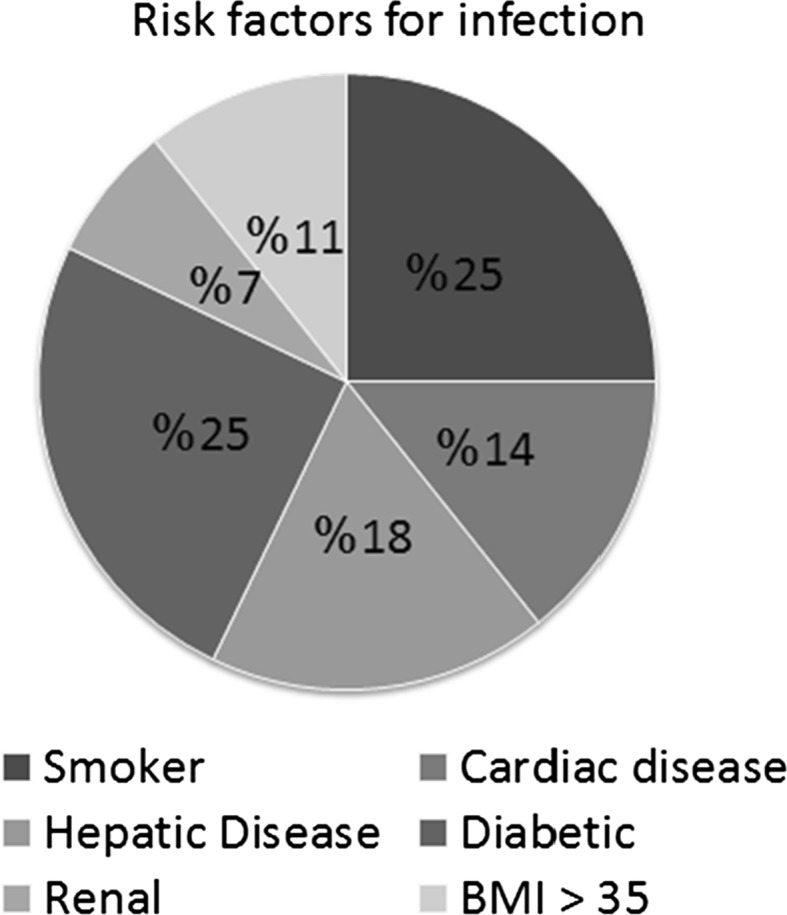
General health risk factors for infection

Twenty-six underwent the planned two-stage hip arthroplasty and one case was lost following the first stage. There were 17 males and 10 females. The average age of the patients was 48.9 years (range 26–74 years) with an average follow up period of 44 months (25–72 months). Twenty-two out of the 27 patients had general health risk factors as summarized in Fig. 1.

The twenty-seven patients were referred to the senior author following failure of previous internal fixation (ten with partially threaded cannulated screws, eight with dynamic hip screws (DHS), three with combined DHS and cancellous screws, five with trochanteric plates and one with DHS and fibular graft). The average time from the original fixation of the fracture to the beginning of the staged protocol for arthroplasty was 9 months (range 5–18 months).

Twenty-three of these patients had multiple operations before the staged arthroplasty (21 patients had debridements, 1 had a revision of fixation, 1 had subtrochanteric valgus osteotomy and 2 had removal of their metal work).

The diagnosis of infection was based on clinical criteria (delayed wound healing, discharging wound, persistent sinus) in addition to laboratory investigations like erythrocyte sedimentation rate (ESR) levels >40 mm/h and C-reactive protein (CRP) levels >10 mg/l. The average ESR at initial presentation was 50.7 during the first hour (range 35–70) and 71.5 during the second hour (range 55–95). The average CRP at initial presentation was 15.9 (range 10–45).

**Fig. 2:**
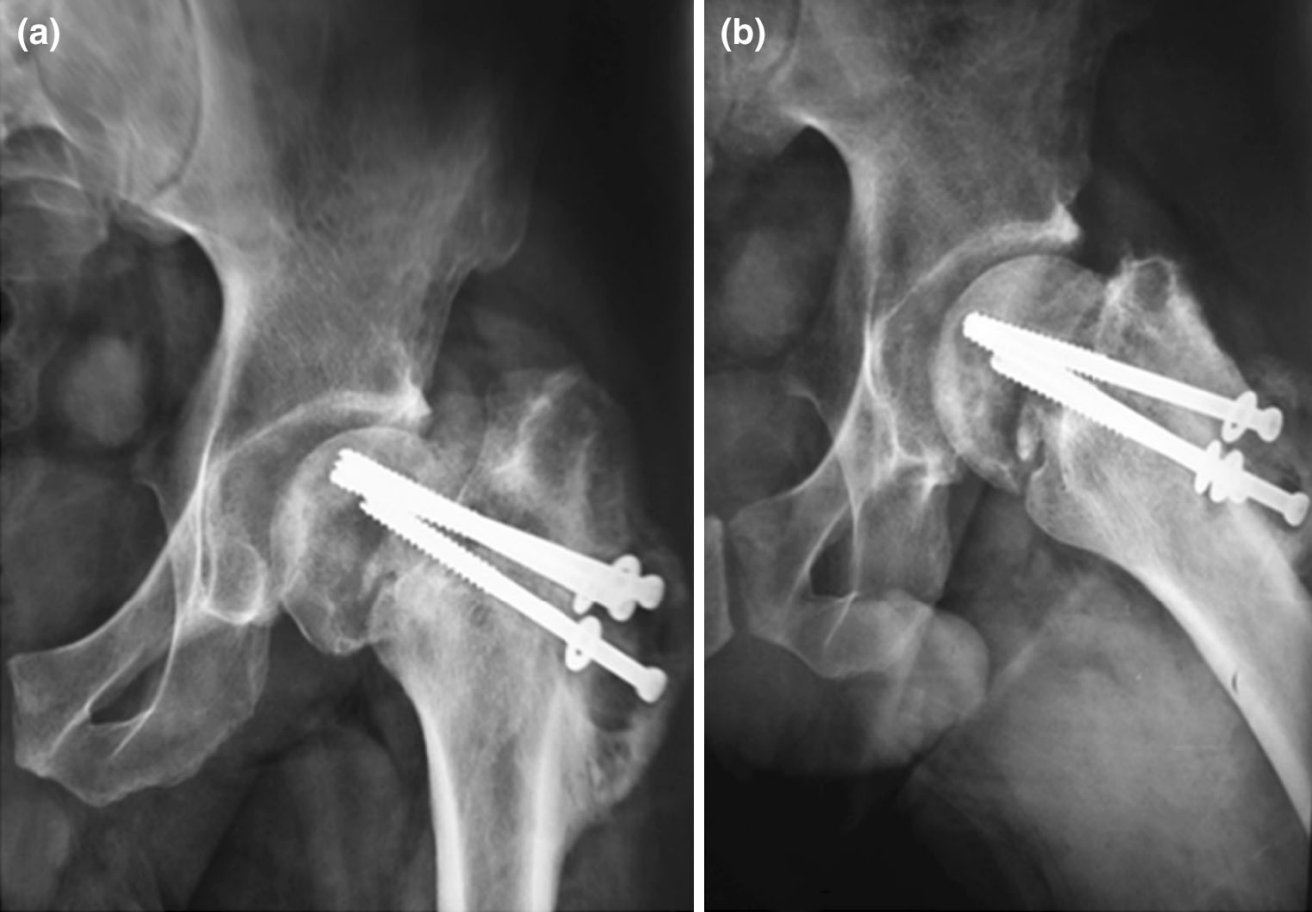
Anteroposterior (**a**) and lateral (**b**) X-rays of the hip in a case of infected non-union of intracapsular fracture showing collapse of the femoral neck in addition to radiological manifestations of infection (osteolysis around the screws and periosteal reaction)

Radiological findings aided in the diagnosis by the presence of osteolysis around the metalwork as well as periosteal reaction (Fig. 2). Failed internal fixation was defined by nonunion at fracture site and/or implant failure.

Identification of the infecting organism was attempted preoperatively. Specimens for culture and sensitivity (C&S) tests were collected from draining sinuses or by preoperative aspiration of the joint fluid. This was done in all cases under aseptic conditions and after having stopped the antibiotics for at least 2 weeks before aspiration.

**Fig. 3:**
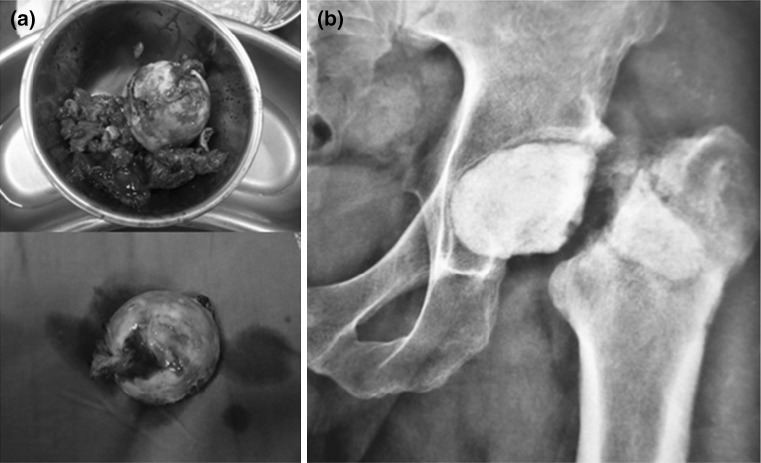
In the first stage **a** radical debridement of the joint removing the hardware plus infected femoral head and soft tissue **b** post-operative X-ray with the spacer in place

The first stage included excision of the sinuses, removal of implants in addition to all necrotic bone and soft tissues. The implants were removed, head and neck of femur were excised and screw tracts were over-reamed (Fig. 3). Only viable tissues were left following extensive debridement. Four to six tissue specimens were collected from different locations: subcutaneous tissues, the hip joint, the femoral canal, screw tracks and were sent for C&S testing.

Custom antibiotic loaded cement spacers were inserted in all cases. Gentamicin loaded bone cement (CMW 1, Depuy, Johnson & Johnson) was used. Additionally, 2 g of antibiotics per cement pack were added in powder form after cement mixing and during molding the spacers (Table 1).

**Table 1:** Types of organism(s) isolated

Type of organism(s)	No. of patients
MRSA & *Klebsiella*	2
*Staph. aureus* & *E. coli*	2
MRSA and *E. coli*	1
*Pseudomonas* & *C. albicans*	1
*Pseudomonas* & *Staph. aureus*	1
MRSA	10
MRSE	4
*E. coli*	3
*Staph. aureus*	2
*Klebsiella*	1

The antibiotic protocol:

The choice of antibiotics added to the cement was determined by the organism isolated in preoperative sensitivity tests (Table 1). When the organism could not be identified by preoperative aspiration or deep swabs from the draining sinuses, a combination of 2 g of Vancomycin and 2 g of Meropenem were added to the cement.

Antibiotics were stopped for 2 weeks prior to the first stage. Having collected tissue specimens, antibiotics were reintroduced during the initial stage of debridement and continued for 6 weeks according to culture and sensitivity results (Table 2). Antibiotics were then stopped for 2 weeks before the second stage and then resumed again after the second stage for 6–12 weeks after implantation.

**Table 2:** Types of antibiotics used

Type of antibiotics used	No. of patients
Vancomycin then Linozolid	10
Vancomycin then Linozolid and Ciprofloxacin	6
Meropenem then Ciprofloxacin	4
Meropenem then Linozolid and Ciprofloxacin	1
Imipenem + Linozolid	2
Imipenem + Diflucan	1
Teicoplanin then Linozolid	1
Vancomycin + Ciprofloxacin then Ciprofloxacin + Linozolid	1

Following the first stage, patients were encouraged to walk with touch weight-bearing. The serum level of CRP and ESR were checked weekly; this is in addition to liver and renal functions every 2 weeks.

Clinical and laboratory parameters were combined to determine the timing of the second stage. An ESR of <30 mm/h (first hour) and CRP <6 mg/l were considered the cut off values for going to the second stage. The average ESR before second stage was 22.9 for the first hour (range 15–50) and 35.3 for the second hour (range 20–70). The average CRP before the second stage was 5.7 (range 3–10).

**Fig. 4:**
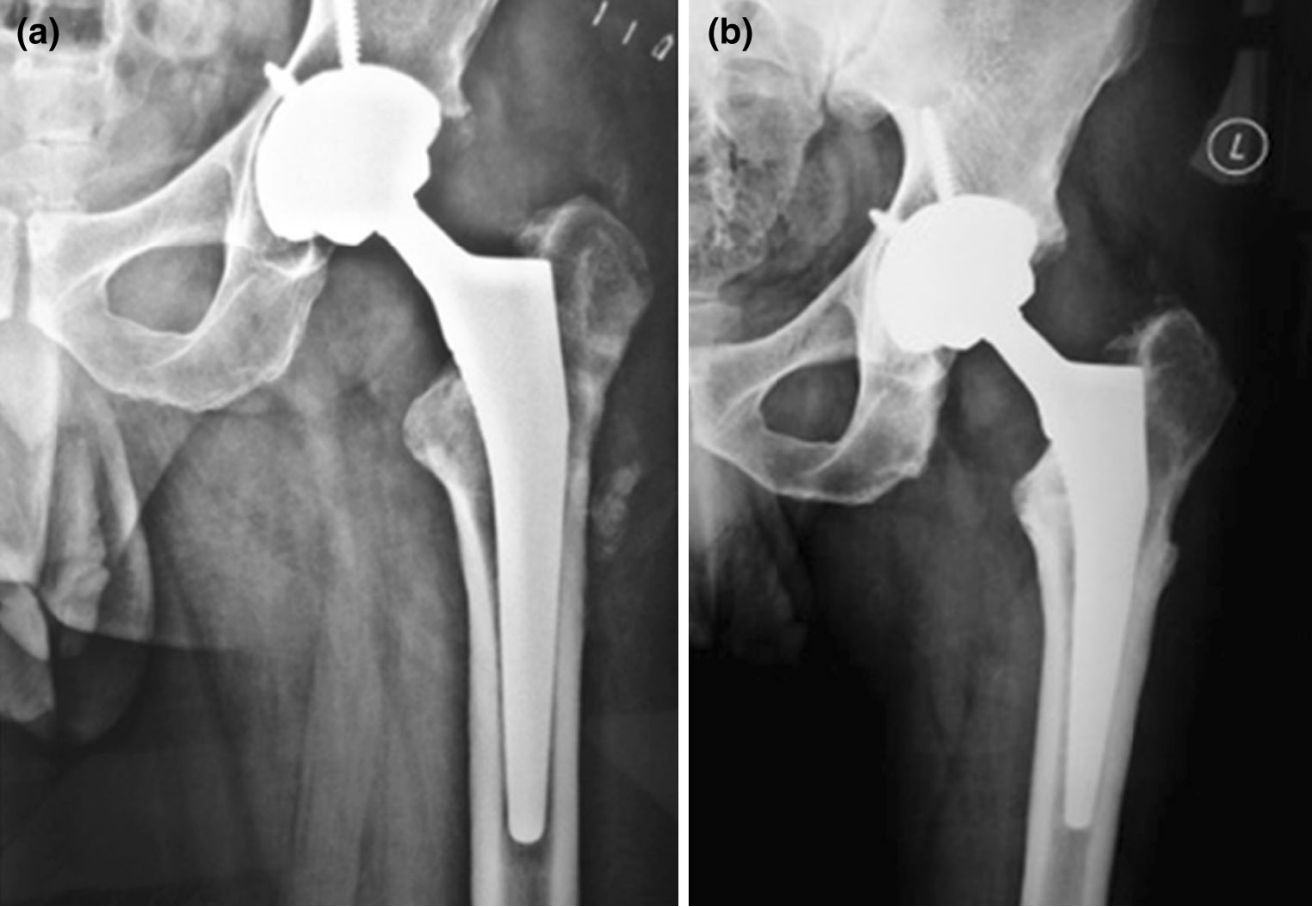
Cementless prosthesis with CoC liner **a** immediate postoperative **b** 3 years follow up with evidence of bone ingrowth and stable implant

The decision about the type of prosthesis used was dependent on the patients’ age, expected level of activity, bony defects as well as the patient’s bone quality (Fig. 4). In case of cemented THR, 1 g of the selected antibiotic powder was added to each 40 g pack of gentamicin loaded bone cement.

**Fig. 5:**
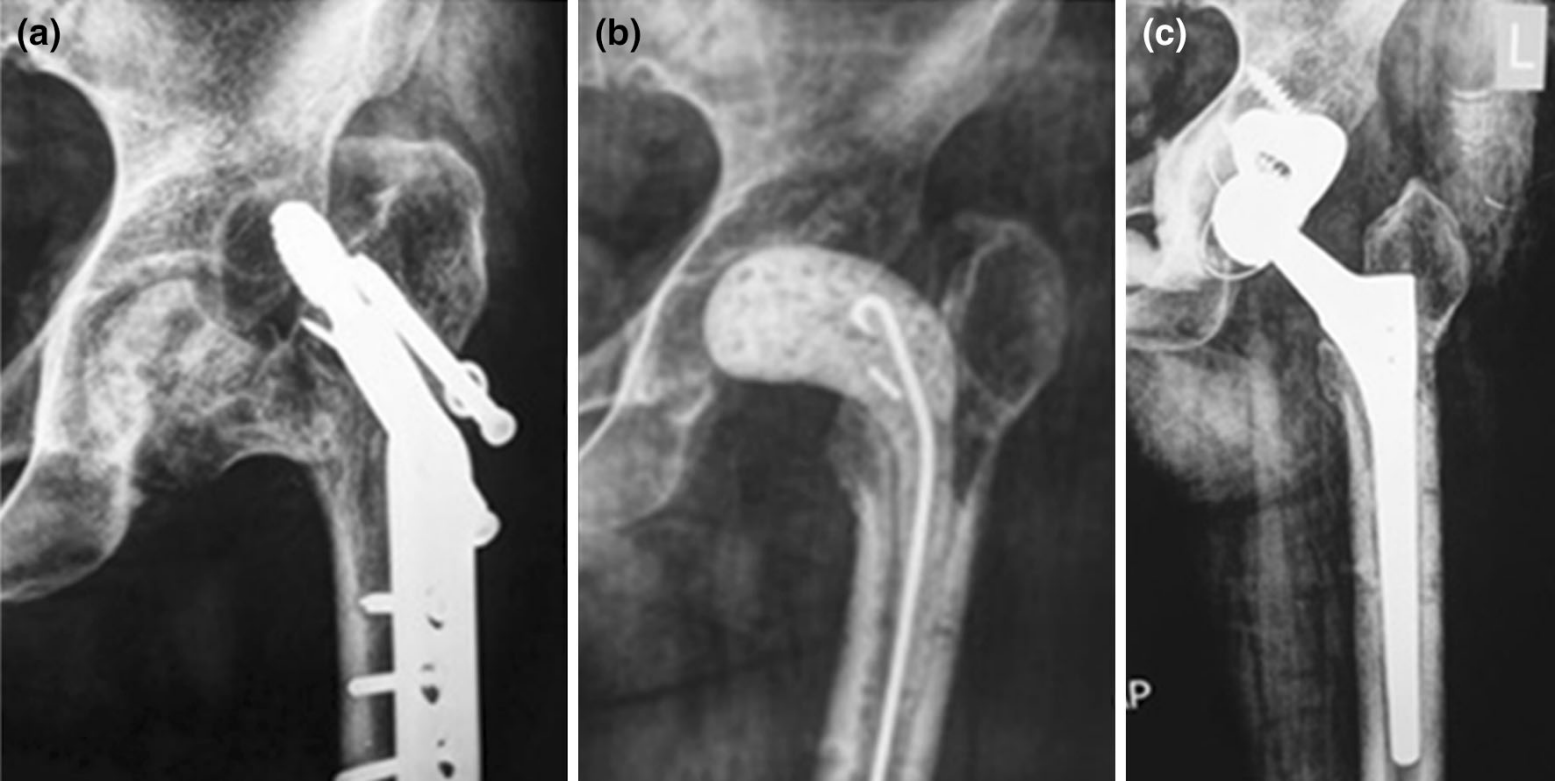
Staged Arthroplasty in a case of infected non-united trochanteric fracture. **a** Failed fixation. **b** After the first stage with the spacer in place. **c** After the second stage using Wagner stem and metal augment

In cases of failed extra-capsular fractures, the bone defects were larger and screw holes along the shafts of the femora were usually encountered. Therefore, long stems either cemented or cementless were employed (Fig. 5).

Various implants were used. On the acetabular side, ten patients had cementless cups (six multiholes Pinnacle cups with ceramic liner and four Novae cementless dual mobility cups). On the other hand, cemented all polyethylene cups were inserted in 16 patients. Eleven of these cemented cups were high cross linked with an inner diameter (ID) of 32 mm; while the remaining five cups were long posterior wall with 28 mm ID.

On the femoral side, 11 patients had cemented stems. Out of these 11, 8 were long CPT double tapered stems and 3 were VerSys cemented stems (Zimmer). Fifteen patients had cementless stems. Eight of these cementless stems were long (four Wagner, Zimmer, and four Carr Depuy, J&J) while the remaining seven were standard fully HA coated stems.

Regarding the bearing surfaces, 17 had metal on polyethylene, 5 had ceramic on polyethylene and 4 had ceramic on ceramic.

Greater trochanter reattachment was done in ten cases. Mersilene tape was used in four cases, metal wires and Ethibond sutures in four and Ethibond trans-osseous sutures in two.

When trochanteric reattachment was performed, patients were allowed only touch weight bearing (WB) for 6 weeks, while avoiding hip flexion beyond 60°. Rehabilitation then progressed to partial WB for another 6 weeks before full WB was allowed at 12 weeks postoperative.

All patients were evaluated at 2, 6, 12 weeks, 6 months and yearly thereafter. The presence of pain at rest as well as on ambulation and the range of motion of the hip were measured. The Harris hip score (HHS) was evaluated preoperatively as well as at 3 and 12 months postoperatively then annually thereafter.

Serial radiographs were used to assess component fixation. Loosening of the acetabular components was defined as implant migration, a complete radiolucent line at the metal bone interface, or breakage of screws [5]. Loosening of the cemented femoral stems was evaluated according to the criteria described by Harris [6], and loosening of the uncemented femoral stems was evaluated according to the system of Engh et al. [7].

Two-tailed Student’s *t* test was used for statistical analysis. Statistical significance was set at 95 % confidence with *P* < 0.05.

## Results

Twenty-seven consecutive patients who had infected nonunion of intra and extra capsular fracture neck of the femur were treated with a staged protocol for total hip arthroplasty. One patient did not complete the planned treatment after the first stage leaving 26 patients for final evaluation. The mean age of this group of patients was 48.9 years (range 26–74 years). The mean duration between the initial fracture fixation and presentation was 16 months (range 8–36 months).

The infecting organism was identified in all patients by the preoperative aspiration in 17 cases, or specimens collected during the first stage in 9 patients. Methicillin resistant staph aureus (MRSA) was the most commonly found organism, occurring in 13/26. However, other organisms were also found like *E*. *coli*, Klebsiella and methicillin-resistant *Staphylococcus epidermidis* (MRSE) (Table 1).

An actively draining sinus was present in nine patients. In seven out of these nine, polymicrobial infection with more than one infecting organism was detected (Table 1).

The average duration between first and second stages was 10 weeks (range 8–16 weeks). All patients were given antibiotics for 6 weeks according to the culture and sensitivity results (Table 2). Following the first stage of debridement, antibiotics in infusion form were given for a minimum of 2 weeks. Antibiotics were then continued for another 4 weeks by IV infusion or oral form when available. These antibiotics were repeated after implantation of the definitive prosthesis for 6–12 weeks.

The staged protocol for THR as a salvage for infected nonunion was successful in eradicating infection on long term follow up in all cases that completed their treatment (Fig. 4). None of the patients had recurrence of infection or further revision surgery at an average 44 months (range 30–72).

The Harris hip score of the patients had improved significantly from 29 ± 6 (mean ± STD) preoperatively to 85 ± 9 after the second definitive surgery (*P* < 0.0001).

None of the patients have had or are awaiting revision. However, one patient had trochanteric detachment with single bout of dislocation 2 weeks from surgery which was treated by closed reduction. Another patient had two bouts of dislocation which were treated by closed reduction and a hip brace.

Few intra-operative complications were recorded. One patient had fissure of the acetabular cup and another one had cortical perforation of the proximal femur. Both fractures were detected intra-operatively and the implant position was corrected.

Two patients had fibrous union of the greater trochanter and another three had leg length discrepancy >1 cm. There were two patients with heterotopic ossification (HO) grade I and II according to Brooke’s classification [8].

Two patients died 30 and 34 months after the second stage from unrelated cardiac and hepatic diseases respectively.

## Discussion

In this series, arthroplasty has been used as an effective treatment for infected non-unions of hip fractures through a staged protocol of management. Both intra and extra-capsular fractures have been included. While there are few studies [9, 10] on the success of THA as a salvage procedure for failed internal fixation of hip, the literature on infected failed fixation is scarce [4].

In a staged protocol for treatment of infected nonunion, an extensive debridement for all infected tissues and identification of the infecting organisms are two essential steps towards success. This extensive debridement usually results in loss of bone stock, fibrosis and sometimes deficient abductor muscles [11–16]. Therefore, revision type of stems may be necessary to achieve distal fixation on the femoral side. In this series 16/26 had long stems. Eight of these long stems were cemented CPT while the other eight were cementless. It was not necessary in this series to use a tumor type implant with proximal femoral replacement, a concept that was adopted by some authors but did not yield a better outcome [17].

Identification of the infecting organism is an important factor in managing these infected cases. When a sinus is present, swabs for C&S test may aid in identifying the organism but cannot substitute cultures performed from tissue specimens collected at the time of debridement [18]. Identification of the infecting organism prior to the first stage of debridement helps in selecting a suitable antibiotic to be added to the cement spacer. Therefore, aspiration was attempted in cases where no sinus tract was present.

The use of antibiotics after the second stage has been a matter of debate. McDonald et al. [19] in a report on the results of 82 two-stage reconstructions that had been performed for the treatment of infection at the site of a hip arthroplasty, recommended at least 4 weeks of intravenous antibiotic therapy when antibiotic-impregnated material is not used. Nestor et al. evaluated 28 patients who had an infection at the site of a hip or knee arthroplasty and demonstrated comparable results between patients who were managed with the implantation of antibiotic-loaded cement beads in conjunction with less than 5 days of parenteral antibiotic therapy and those who were managed with 6 weeks of conventional intravenous antibiotic therapy [20].

In this series, high doses of antibiotics were delivered through the antibiotic loaded cement spacers in addition to systemic administration of IV or oral treatment. Antibiotics continued to be used for 6 weeks after the first stage then for another 6–12 weeks after implantation. This protocol of management proved to be successful in controlling infection with no relapse at the latest follow up.

It can be argued that a single stage of debridement and implantation may reduce the cost of treatment and allow early mobilization of patients. However, previous reports on THR following osteosynthesis have higher rates of infection than primary THR for arthritis [21]. Additionally, infection in these cases involves different tissue planes within and outside the hip space that would make satisfactory debridement in all cases difficult to achieve especially when a draining sinus is present. Finally, identification of the infecting organism is an essential step for a successful single stage arthroplasty; a pre-requisite that could only be achieved in some but not all cases.

Because of high incidence of instability that is usually seen in these cases of difficult primary THR, large head diameter was used whenever there was trochanteric detachment or questionable abductor function. Thirty-two millimetre head diameter was used in 11/26. The 36 mm head diameter was used in 4/26 patients while the dual mobility heads were inserted in another 4/26. This would leave only 7 out of 26 who had 28 mm head diameter. It remains that even though large head diameter was selected for 73 % of the patients, dislocation was encountered in two patients.

Instability post THR for fractures is considerably higher than that when THR is performed for arthritis. This is particularly true when managing trochanteric fractures [22–24]. Many factors may be contributing to this high incidence of post-operative instability from abductor deficiency, trochanteric displacement to cognitive dysfunction in this elderly population. Dual mobility heads may present a good alternative for these difficult cases [25, 26].

Reattachment of the trochanter or augmentation of a weak attachment was performed regularly in extracapsular fracture types. Only, two patients had trochanteric nonunion with displaced trochanter.

The ESR and serum CRP level are not specific measures of infection [27]. However, these tests are sensitive, readily available, most useful and prognostically significant when they are monitored serially [14]. Hence, the method of using the ESR and serum CRP level to guide the timing of reimplantation is a more practical approach than selecting a fixed interval between procedures because the virulence of the causative pathogen, the severity of infection, and the host response to antimicrobial treatment may vary greatly among individuals [4, 13].

In all patients, custom-made cement spacers were used in the interim period. The spacer maintains soft tissue tension, avoids shortening, improves gait pattern, and provides local release of antibiotics [16, 28]. Dislocation of the spacer is reported to be a common complication, with rates ranging from 1.8 to 18 % in various studies [4, 11, 29]. However, it was more related to the geometry of the spacer with geometric mismatch being a common cause for the dislocation. In this series, efforts were made to match the shape and size of the femoral head and hence problems related to the use of the spacer were minimized.

Hip function for patients included in this series showed a significant improvement in the HHS from pre to postoperative. This was found to be comparable with primary hip arthroplasty for femoral neck fractures. Narayan et al. [30] have found the average HHS for patients of femoral neck fractures treated with total hip arthroplasty to be 83.8, while Patel et al. [31] have also reported similar results in their group of patients.

The only other series comparable to the present series was that of Hseih et al. [4] which has discussed the use of hip arthroplasty in infected intertrochanteric fractures. Similar results were obtained in this series with all the patients having significant improvement in their functional scores. In their series, all patients were treated with two-stage revision with either cement beads or cemented spacer in the interim period.

The strengths of the present study include a relatively good number of consecutive patients of this difficult problem with a high rate of follow up. Additionally, this study includes not only intertrochanteric fractures but also transcervical fractures.

The limitation of this study is the lack of a control group and relatively medium term follow up. Although there were no deep re-infections in these patients at the last follow up, it may still be early to comment on the long term outcome of the implants.

To conclude, staged THA was very effective in restoring function in patients with deep hip sepsis after failed treatment of hip fractures. Complete and radical debridement in the first stage, proper antibiotic therapy as well as strict weekly based clinical and laboratory follow ups helped in achieving a desirable outcome. The use of the antibiotic loaded cement spacer was a very inexpensive and effective method.
